# MicroRNA-223 delivered by platelet-derived microvesicles promotes lung cancer cell invasion via targeting tumor suppressor EPB41L3

**DOI:** 10.1186/s12943-015-0327-z

**Published:** 2015-03-11

**Authors:** Hongwei Liang, Xin Yan, Yi Pan, Yongsheng Wang, Nan Wang, Limin Li, Yuan Liu, Xi Chen, Chen-Yu Zhang, Hongwei Gu, Ke Zen

**Affiliations:** From State Key Laboratory of Pharmaceutical Biotechnology, Nanjing University School of Life Sciences, Jiangsu Engineering Research Center for MicroRNA Biology and Biotechnology, Nanjing, Jiangsu 210093 China; The Comprehensive Cancer Center of Drum Tower Hospital affiliated to Medical School of Nanjing University & Clinical Cancer Institute of Nanjing University, Nanjing, Jiangsu 210008 China; Department of Respiratory Medicine, Nanjing Drum Tower Hospital Affiliated to Medical School of Nanjing University, 321 Zhongshan Road, Nanjing, 210008 Jiangsu Province China; Department of Biology, Georgia State University, Atlanta, GA 30303 USA

**Keywords:** Platelet, Microvesicles, MicroR-223, Lung cancer

## Abstract

**Background:**

Patients with hematogenous metastatic lung cancer displayed significantly increased platelet count and aggregation compared to lung cancer patients without hematogenous metastasis. The mechanism underlying the correlation between the lung cancer hematogenous metastasis and platelet activation remains unknown.

**Results:**

In the present study, we explored the role of microRNA-223 (miR-223) derived from platelets in modulating lung cancer cell invasion. Our results demonstrated that platelets from NSCLC patients contain higher level of miR-223 than that from healthy subjects. The concentration of miR-223 in the platelet-secreted microvesicles (P-MVs) from NSCLC patients was also increased compared to that from healthy subjects. Incubation of human lung cancer A549 cells with P-MVs resulted in rapid delivery of miR-223 into A549 cells, in which platelet miR-223 targeted EPB41L3 and thus promoted A549 cell invasion. The effect of P-MVs on reducing EPB41L3 in A549 cells but promoting tumor cell invasion could be largely abolished by depletion of miR-223 via transfection with miR-223 antagomir. The role of EPB41L3 in inhibiting A549 cell invasion was further validated by directly downregulating EPB41L3 via transfecting cells with EPB41L3 siRNA or miR-223 mimic.

**Conclusions:**

Our study demonstrates for the first time that platelet-secreted miR-223 via P-MVs can promote lung cancer cell invasion via targeting tumor suppressor EPB41L3.

**Electronic supplementary material:**

The online version of this article (doi:10.1186/s12943-015-0327-z) contains supplementary material, which is available to authorized users.

## Background

Lung cancer is the most common cause of cancer-related death in the world. Non-small cell lung cancer (NSCLC) comprises approximately 80% of lung cancers [1]. Hematogenous and lymphatic metastases are the most common metastatic pathways for lung cancers. At the earlier stages, lung cancer cell metastasizes through the lymphatic system. As hematogenous metastasis often occurs when lung cancer develops into an advanced stage, hematogenous metastasis is generally associated with a poor prognosis of lung cancer patients and an accelerated mortality rate of lung cancer patients [2]. It has previously demonstrated that, compared with healthy controls and lung cancer patients without hematogenous metastasis, patients with hematogenous metastatic lung cancer displayed significantly increased platelet count and aggregation [3]. Close correlation between the hematogenous metastasis of lung cancer and platelet activation was also reported by others [4]. However, the mechanism underlying the role of platelets in modulating the hematogenous metastasis of lung cancer remains unclear.

It has been well documented that activated platelets release large amount of microvesicles (MVs) [5,6]. Platelets are major sources of MVs in peripheral bloodstream as two-thirds of peripheral blood MVs is likely derived from platelets [7]. In response to the stimuli of various inflammatory factors or under many disease states such as sepsis [8,9], thrombocytopenia [10], arterial thrombosis [11], thrombotic thrombocytopenia [12], uremia [13], malignancy [14] and rheumatoid arthritis [15], platelets can release more MVs. Similar to MVs derived from other cells, platelet MVs (P-MVs) bear platelet surface receptors/ligands and have the potential to selectively interact with specific target cells. Increasing evidence demonstrates that P-MVs play a role in coagulation, angiogenesis, the metastatic spread of lung cancer [9], and the immune response of hematopoietic, endothelial, and monocytic cells [16,17]. P-MVs have also been implicated in the pathogenesis of atherosclerosis as well as the regulation of angiogenesis [16,18]. These findings suggest that, without the physical contact with the target cells, platelets can affect the functional state of the cells through releasing P-MVs. However, despite the extensive studies regarding P-MVs in various physiological and pathological processes, the mechanism that governs the role of P-MVs in target cells, particularly cancer cells, remains to be further explored.

MicroRNAs (miRNAs) are a class of noncoding RNAs that post-transcriptionally regulate gene expression in plants and animals [19]. Through guiding the binding of the RNA-induced silencing complex (RISC) to complementary sequences in the 3-untranslated region (UTR) or the open reading frame (ORF) of target mRNA molecules, miRNAs either degrade mRNA or block the gene translation. Accumulating evidence has demonstrated that miRNAs play a key role in the cellular processes of differentiation, proliferation, maturation, and apoptosis. Our recent studies have demonstrated that miRNAs are stably expressed in animal serum/plasma and that their unique expression patterns serve as “fingerprints” of various diseases [20]. Mechanistic studies further suggest that cells can selectively secret miRNAs via MVs in response to various stimuli and that these MV-encapsulated miRNAs are associated with Argonaute 2 (AGO2) complexes [21,22]. Secreted miRNAs in MVs can be efficiently delivered into target cells, in which they silence their target genes and thus affect recipient cell function [21,23]. Therefore, cell-secreted miRNAs in MVs can serve as a novel class of signaling molecules to remotely mediate intercellular communication.

Recent studies have shown that anucleate platelets also contain abundant miRNAs [24-27], though the biogenesis pathway of miRNAs in anucleate platelets remains unclear. In addition, platelets have been shown to express certain miRNA processing machinery including Dicer, RNA-binding protein 2 and AGO2 [24], implying that platelet miRNA may have biological functions and that platelets may be able to process pre-miRNA into mature miRNA. Diehl et al. [28] reported that miRNAs, including miR-19, miR-21, miR-126, miR-133, miR-146 and miR-223, could be detected in P-MVs, suggesting that platelets can secrete their miRNAs through P-MVs. Delivery of functional platelet miRNAs into endothelial cells via P-MVs has be reported recently [29,30]. Although many mRNAs have been predicted to be targets of these platelet miRNAs, the function of platelet miRNAs particularly the miRNAs stored in P-MVs, has yet to be shown. The work by Gidlof and co-workers [30] provided the first evidence that activated platelets can release functional miRNA, which can be taken up by endothelial cells and regulate endothelial intercellular adhesion molecules 1 (ICAM1) expression, suggesting that delivery of functional platelet miRNAs into vascular endothelial cells by P-MVs can play a critical role in modulating vascular endothelial inflammatory responses. Recently, we reported that P-MVs could effectively deliver miR-223 into human umbilical vein endothelial cells (HUVECs), in which platelet miR-223 targeted IGF-1R and promoted HUVEC apoptosis induced by advanced glycation end products (AGEs) [31]. Given that miR-223 is the most abundant miRNA in the platelets and P-MVs [31,32] and its expression is deregulated in many types of cancer [33-36], it is considered as a member of an emerging family of tumor-promoting miRNAs called oncomiRs. It has been reported that miR-223 could promote breast cancer invasiveness by suppressing Mef2c (Myocyte enhancer factor 2c) [33]. Li et al. [34] examined miRNAs in several human gastric cell lines and showed that miR-223 is specifically overexpressed in metastatic gastric cells and stimulates tumor cell invasion. It has been also reported that miR-223 directly target the 3′UTR of erythrocyte membrane protein band 4.1-like 3 (EPB41L3) [35] and is the most upregulated miRNA in recurrent tumors [36].

In the present study, we explored the role of miR-223 derived from anucleate platelets in modulating lung cancer cell invasion. Our results demonstrated that platelets from NSCLC patients contain higher level of miR-223 than that from healthy subjects. The concentration of miR-223 in the P-MVs from NSCLC patients was also increased compared to that from healthy subjects. Furthermore, we showed that P-MVs could effectively deliver miR-223 into human lung cancer cells A549, in which platelet miR-223 targeted EPB41L3 and thus promoted A549 invasion.

## Results

### Levels of miR-223 and pre-miR-223 in platelets from NSCLC patients were significantly increased compared to that from healthy volunteers

**Figure 1:**
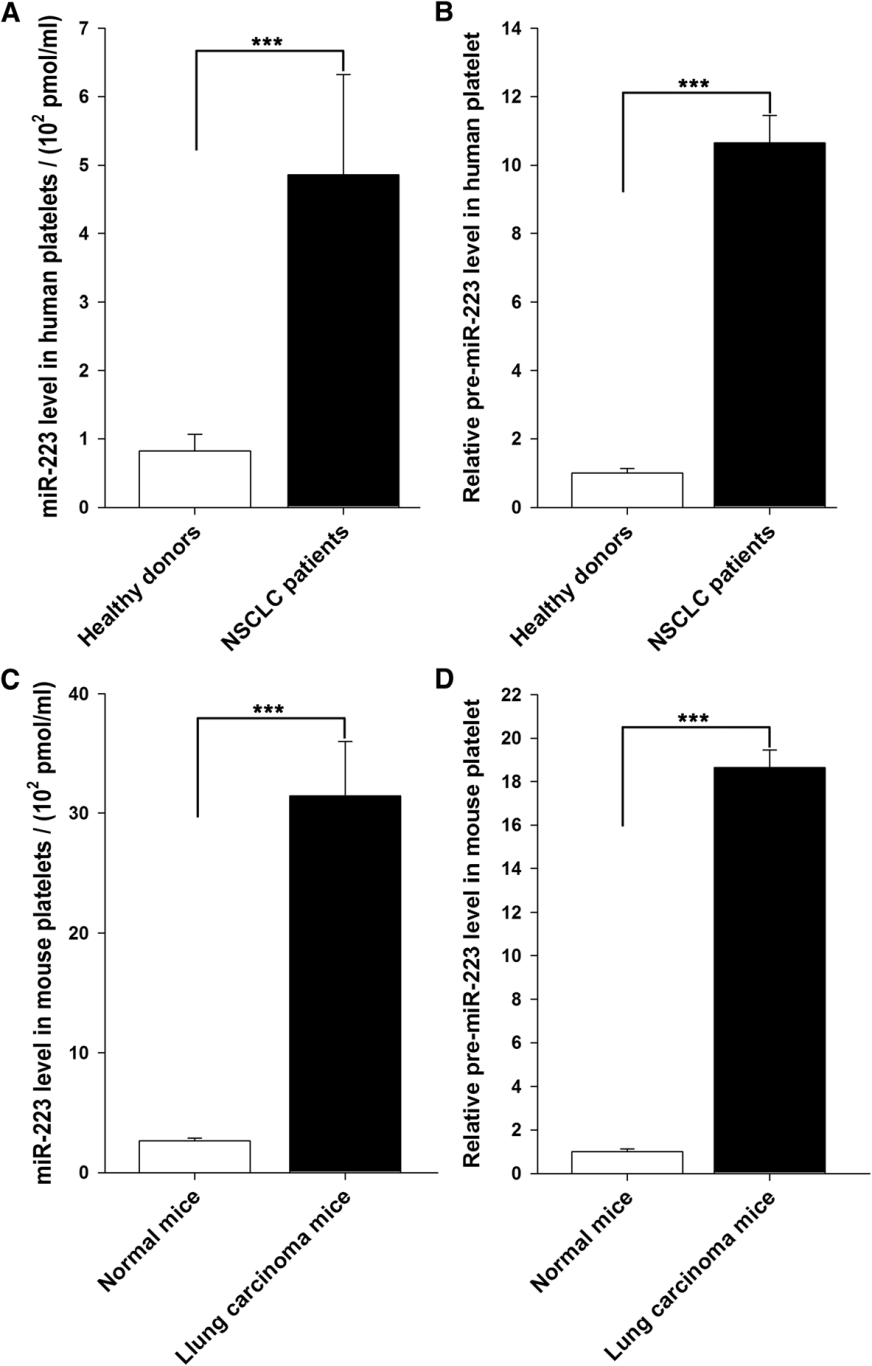
**The levels of miR-223 and pre-miR-223 in platelets isolated from NSCLC patients or Lewis lung carcinoma mice. (A)** The absolute levels of miR-223 in the platelet from healthy volunteers and NSCLC patients detected by qRT-PCR. **(B)** The relative levels of pre-miR-223 in the platelet from healthy volunteers and NSCLC patients detected by qRT-PCR. **(C)** The absolute levels of miR-223 in the platelet of Lewis lung carcinoma mice detected by qRT-PCR. **(D)** The relative levels of pre-miR-223 in the platelet of Lewis lung carcinoma mice detected by qRT-PCR. The levels of miR-223 and pre-miR-223 were assessed by qRT-PCR and normalized against blood volume. Results are presented as mean ± SEM of five independent experiments (*, *P* < 0.05, **, *P* < 0.01,***, *P* < 0.001).

The clinical parameters of platelets of 20 cases of diagnosed NSCLC patients and 20 cases of diagnosed healthy donors were determined at the Jinling Hospital (Nanjing, China) (Table 1). As the results of previous studies [3,4], we found the platelet numbers (PLT) and platelet hematocrit (PCT) in NSCLC patients were significantly higher than healthy donors, while the mean platelet volume (MPV) and platelet distribution width (PDW) were no different between NSCLC patients and healthy donors. As miR-223 is the most abundant miRNA in the platelets [25,31,37], we compared the miR-223 level in the platelets from NSCLC patients with that from healthy volunteers using TaqMan probe-based qRT-PCR assay. As shown in Figure 1A, miR-223 in the platelet of per milliliter blood from NSCLC patients increased about 10-fold than healthy volunteers. We found that the miR-223 level increased from 3.86 × 10^−8^ fM of healthy volunteers to 1.80 × 10^−6^ fM of NSCLC patients per platelet (Additional file 1: Figure S1). As anucleate platelets have no nuclear microprocessor components such as Drosha and DGCR8 [25], the *de novo* biogenesis of miRNA should be not considered as a mechanism underlying the upregulation of miRNAs in platelets. However, Landry et al. [24] have shown that platelets have Dicer and AGO2 and are capable of processing pre-miRNAs into mature miRNAs. The up-regulation of miRNAs in platelets of NSCLC patients may be due to the enhanced maturation of pre-miRNAs. We thus assayed the levels of pre-miR-223 in platelets. The cellular levels of pre-miR-223 were detected by qRT-PCR using the primer listed in Additional file 1: Table S1. As shown in Figure 1B, the levels of pre-miR-223 in the platelets of NSCLC patients were rapidly increased compared to platelets of healthy volunteers, which is inversely correlated to the up-regulation of miR-223 in platelets of NSCLC patients. Taken together, these results implicate that the levels of miR-223 and pre-miR-223 in the platelets of NSCLC patients relatively increased compared to healthy volunteers. By intravenous injection, we successfully established a mouse model of Lewis lung carcinoma in situ (Additional file 1: Figure S2) and also found that the concentration of miR-223 and pre-miR-223 in platelet from mouse peripheral bloodstream was significantly elevated compared to normal mice (Figure 1C and D).

**Table 1:** **Demography and clinical features of NSCLC patients and healthy subjects**

**Variable**		**Normal volunters (N = 20)**		**NSCLC patients (N = 20)**		**p-value**
		**NO.**	**%**	**NO.**	**%**	**(LC vs Health)**
Average age (years)		60.6 ± 8.93		63.57 ± 10.26		0.535
Sex (male)		14	70	12	60	0.612
Smoking status	current	9	45	10	50	0.798
	Ever	2	10	3	15	
	Never	9	45	7	35	
Histological type	Adenocarinoma			6	30	
	Squamous cell carcinoma			10	50	
	Large cell carcinoma			4	20	
Stage	I/II			5	25	
	III			6	30	
	IV			9	45	

Statistical comparison was performed by using Student’s t-test.

### MiR-223 significantly increased in MVs derived from platelets of NSCLC patients

**Figure 2:**
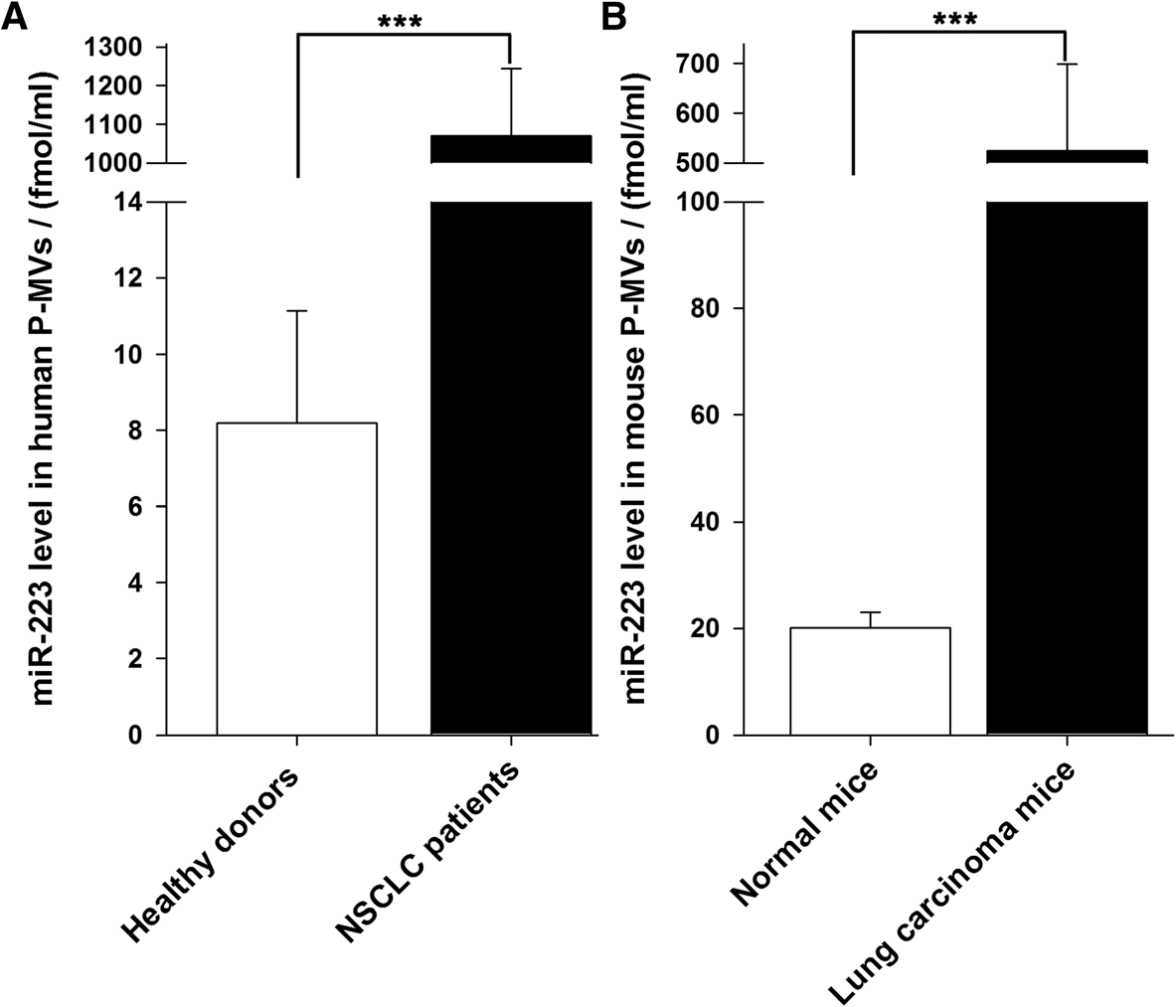
**The level of miR-223 in P-MVs isolated from NSCLC patients and Lewis lung carcinoma mice. (A)** The absolute levels of miRNAs in the P-MVs isolated from healthy volunteers and NSCLC patients detected by qRT-PCR. **(B)** The absolute levels of miRNAs in the P-MVs isolated from control mice and Lewis lung carcinoma mice detected by qRT-PCR. Results are presented as mean ± SEM of five independent experiments (*, *P* < 0.05, **, *P* < 0.01, ***, *P* < 0.001).

It has been widely reported that activated platelets release large amount of microvesicles (P-MVs), especially after stimulation with agonists including thrombin or exposure to high-stress shear forces [13,31,38,39]. In fact, previous studies suggest that the majority of MVs in peripheral bloodstream are derived from platelets, while mononuclear phagocytes are the second most abundant population [40,41]. Numerous studies show that P-MVs were substantially increased in tumor patients compared to the healthy persons [21,42,43]. We measured the levels of miR-223 in P-MVs and found that not only P-MVs contained considerable levels of miR-223, but also the levels of miR-223 in P-MVs was significantly increased in NSCLC patients compared to healthy volunteers (Figure 2A). Interestingly, we also found that the concentration of miR-223 in circulating MVs from mouse peripheral bloodstream was significantly elevated compared to normal mice (Figure 2B). Because P-MVs generally account for two-thirds of circulating MVs in peripheral bloodstream, this result suggests that P-MVs from Lewis lung carcinoma mice contain a higher level of miR-223 than normal mice.

### P-MVs effectively deliver miR-223 into the recipient A549 cells

**Figure 3:**
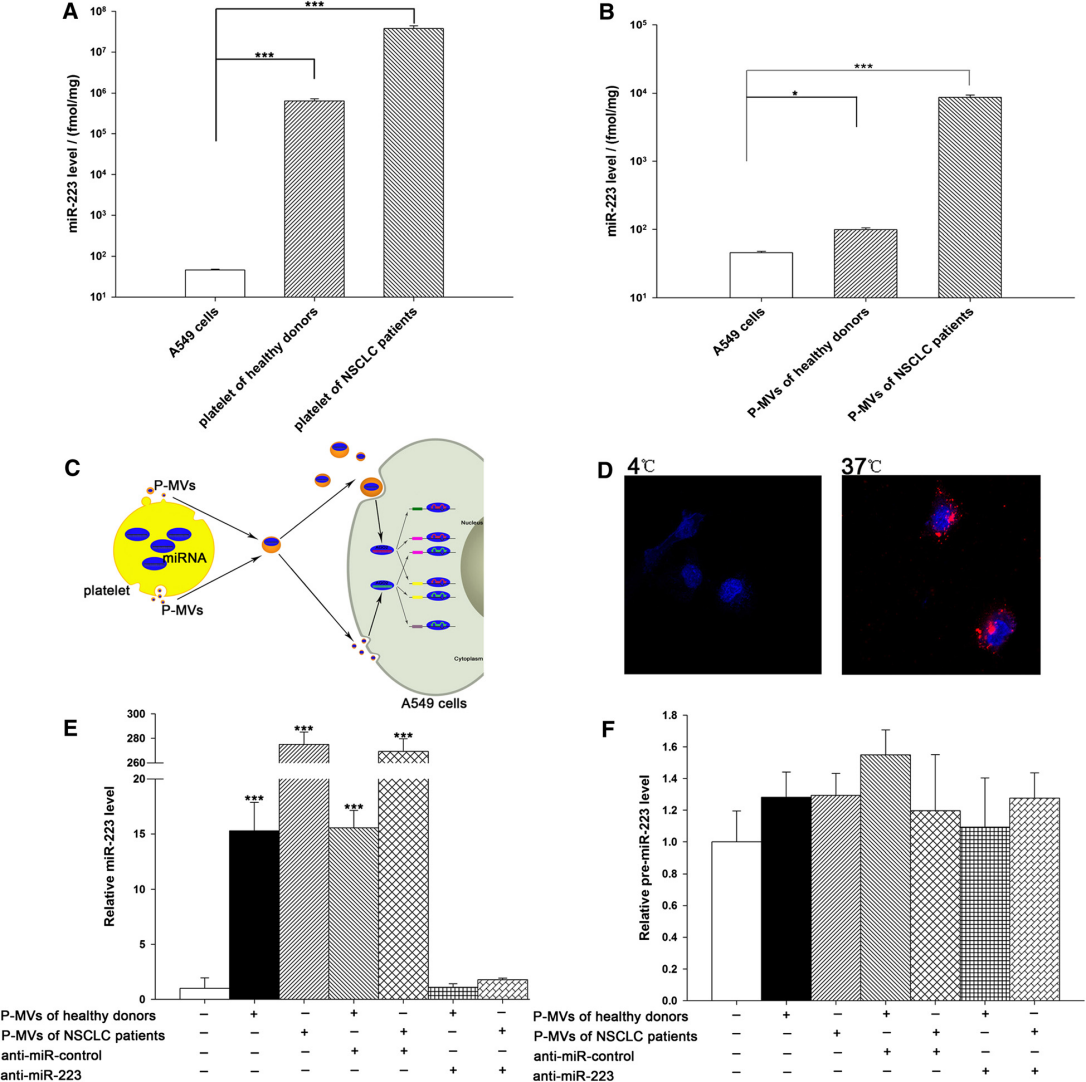
**Effective delivery of platelet miR-223 into A549 cells by P-MVs. (A)** Comparison of the absolute levels of miR-223 in platelets and A549 cells. The level of miR-223 was assessed by qRT-PCR and normalized against the amount of total RNA. **(B)** Comparison of the absolute levels of miR-223 in P-MVs and A549 cells. The level of miR-223 was assessed by qRT-PCR and normalized against the amount of total RNA. **(C)** Flow chart of the experimental design. **(D)** Confocal microscopy image of the internalization of fluorescently labeled MVs into A549 cells. Platelets were labeled with DiI-C_16_ (red). After incubation for 4 h, the supernatants were collected and centrifuged to harvest P-MVs. The isolated P-MVs were re-suspended in RPMI 1640 medium supplemented with 10% FBS and incubated with A549 cells at 4°C or 37°C, respectively. After incubation for 2 h, A549 cells were washed, fixed, and observed under confocal microscopy. (E-F) Quantitative RT-PCR analysis of miR-223 **(E)** and pre-miR-223 **(F)** expression level in A549 cells treated without P-MVs (mock) or with P-MVs from various sources for 24 h. Data are presented as the mean ± SEM of five independent experiments (*, *P* < 0.05, **, *P* < 0.01,***, *P* < 0.001).

Recently, the studies by us [31] and Laffont et al. [32] have demonstrated that platelet secreted MVs can effectively deliver miRNAs into the recipient cells to modulate the expression of their target genes. To determine the potential biological functions of the miRNA-containing P-MVs, we studied the delivery of miR-223 into A549 cells via P-MVs. First, the comparison of miR-223 levels in A549 cells, platelets of healthy volunteers and NSCLC patients, and P-MVs by TaqMan probe-based qRT-PCR assay clearly showed that miR-223 level in platelets (Figure 3A) and P-MVs (Figure 3B) was much higher than that in A549 cells, especially, the miR-223 level in P-MVs of NSCLC patients is about 100-fold more than A549 cells. Second, we collected P-MVs released from platelets and incubated the P-MVs with cultured A549 cells, as depicted in Figure 3C. For tracing the P-MVs in A549 cells, we labeled the P-MVs with DiI-C_16_ and then detected the localization of fluorescent P-MVs in A549 cells after incubation at different temperatures. As shown in Figure 3D, fluorescent P-MVs were found rapidly entering the cultured A549 cells after incubation at 37°C (in red), whereas the internalization of P-MVs into A549 cells was blocked at 4°C. This result showed that internalization of P-MVs into recipient cells is an active process. In agreement with this observation, incubation of A549 cells with P-MVs at 37°C strongly increased miR-223 level, and this elevation could be largely abolished by co-transfecting A549 cells with anti-miR-223 antisense oligonucleotide (ASO) (Figure 3E). In contrast, the pre-miR-223 level in the recipient A549 cells was not altered by incubation with P-MVs (Figure 3F), suggesting that the elevation of miR-223 level in A549 cells is not due to *de novo* miRNA biosynthesis but derived from P-MV delivery. More interestingly, although the incubation with P-MVs of healthy volunteers could increase the miR-223 level in the A549 cells more than 20-fold, incubation with the P-MVs of NSCLC patients further increased the miR-223 level in the A549 cells more than 200-fold (Figure 3E).

### Platelet miR-223 promotes A549 cells invasion via targeting EPB41L3

**Figure 4:**
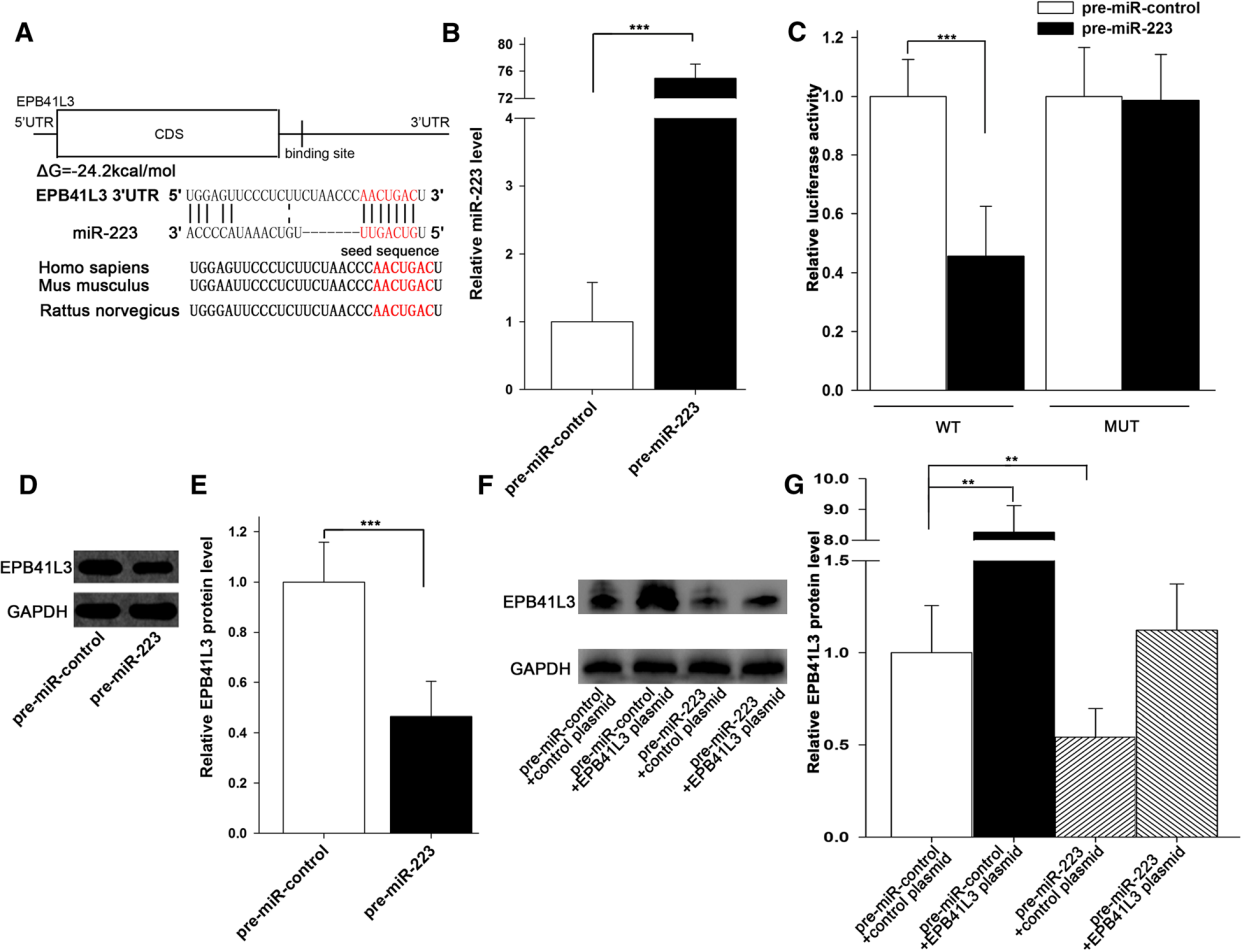
**Direct regulation of EPB41L3 expression by miR-223. (A)** Schematic depicting the hypothetical duplexes formed by the interactions between the binding sites in the EPB41L3 3′-UTR (top) and miR-223 (bottom). The predicted free energy value of each hybrid is indicated. The seed recognition sites are denoted, and all nucleotides in these regions are highly conserved across species, including human, mouse and rat. **(B)** Quantitative RT-PCR analysis of miR-223 levels in A549 cells treated with pre-miR-control and pre-miR-223. **(C)** Firefly luciferase reporters containing wild-type (WT) or mutant (MUT) miR-223 binding sites in the EPB41L3 3′-UTR were co-transfected into A549 cells along with pre-miR-control, or pre-miR-223. he cells were assayed using a luciferase assay kit 24 h post-transfection. **(D-E)** Western blotting analysis of EPB41L3 protein levels in A549 cells treated with pre-miR-control, or pre-miR-223. D: representative image; E: quantitative analysis. **(F-G)** Western blotting analysis of EPB41L3 protein levels in A549 cells treated with pre-miR-control plus control plasmid, pre-miR-control plus EPB41L3 plasmid, pre-miR-223 plus control plasmid, or pre-miR-223 plus EPB41L3 plasmid. F: representative image; G: quantitative analysis. Data are presented as the mean ± SEM of five independent experiments (*, *P* < 0.05, **, *P* < 0.01,***, *P* < 0.001).

To determine whether exogenous miR-223 delivered by P-MVs has a biological function in A549 cells, we analyzed the potential target genes of miR-223 using three computer-aided algorithms, TargetScan, miRanda, and PicTar. As shown in Figure 4A, we predicted EPB41L3 as a target gene of miR-223. As the previous study [34], overexpression of miR-223 in A549 cells via transfection of miR-223 mimic (Figure 4B) clearly showed that miR-223 strongly reduced protein level (Figure 4D and E). As expected, luciferase activity was also markedly reduced in the cells transfected with pre-miR-223 (Figure 4C). Furthermore, we introduced point mutations into the corresponding complementary sites in the 3′-UTR of EPB41L3 to eliminate the predicted miR-223 binding sites. This mutated luciferase reporter was unaffected by overexpression miR-223 (Figure 4C). This finding suggested that the binding site strongly contribute to the interaction between miR-223 and EPB41L3 mRNA. Moreover, compared with cells transfected with pre-miR-223 alone, those transfected with both pre-miR-223 and the EPB41L3-expressing plasmid lack of the miR-223-responsive 3′-UTR, exhibited higher EPB41L3 protein levels (Figure 4F and G), suggesting that miR-223-resistant EPB41L3 is sufficient to rescue the suppression of EPB41L3 by miR-223. In conclusion, our results demonstrated that miR-223 directly binds to the 3′-UTR of the EPB41L3 mRNA transcript and inhibits EPB41L3 translation.

**Figure 5:**
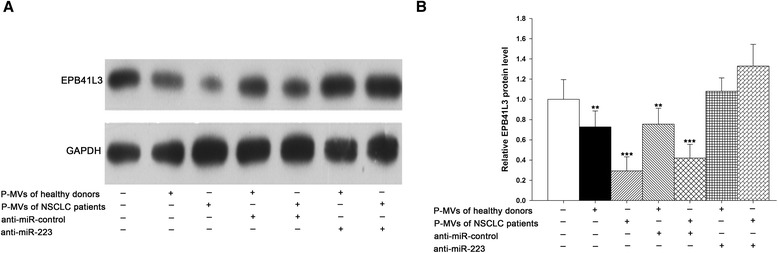
**Exogenous miR-223 delivered by P-MVs reduced EPB41L3 protein expression in A549 cells. (A-B)** EPB41L3 protein levels of A549 cells treated without P-MVs (Mock), or with P-MVs from various sources for 24 h. **A**: representative image; **B**: quantitative analysis. Data are presented as the mean ± SEM of five independent experiments (*, *P* < 0.05, **, *P* < 0.01,***, *P* < 0.001).

We next determined whether the exogenous miR-223 delivered by P-MVs could affect the level of EPB41L3 in the recipient A549 cells. Western blot analysis demonstrated that incubation of A549 cells with P-MVs significantly decreased the levels of cellular EPB41L3 protein, and this reduction of EPB41L3 protein levels by P-MVs was largely abolished by co-transfection with the anti-miR-223 ASO (Figure 5A and B). These results confirmed the role of miR-223 delivered by P-MVs in reducing EPB41L3 levels in the A549 cells. More interestingly, the P-MVs of NSCLC patients significantly suppressed the EPB41L3 protein level in A549 cells compared to P-MVs of healthy volunteers.

EPB41L3, a member of the band 4.1 family of cytoskeletal proteins, is considered to function as a linker between the actin cytoskeleton and transmembrane proteins [44]. EPB41L3 can regulate cell shape, cell–cell and cell–substrate adhesion, cell motility, and take part in the organization of the actin cytoskeleton [45]. Decreased motility and increased adhesion due to EPB41L3 re/overexpression has been shown in several different cancer cell lines [45,46]. Furthermore, siRNA knockdown of EPB41L3 in non-metastatic cell lines stimulated invasion and the loss of actin stress fibers [47]. As detachment of tumor cells from the primary tumor and invasion into the surrounding tissue is the first step of the metastatic cascade [48], these functions of EPB41L3 indicate a possible metastasis suppressor activity [49].

**Figure 6:**
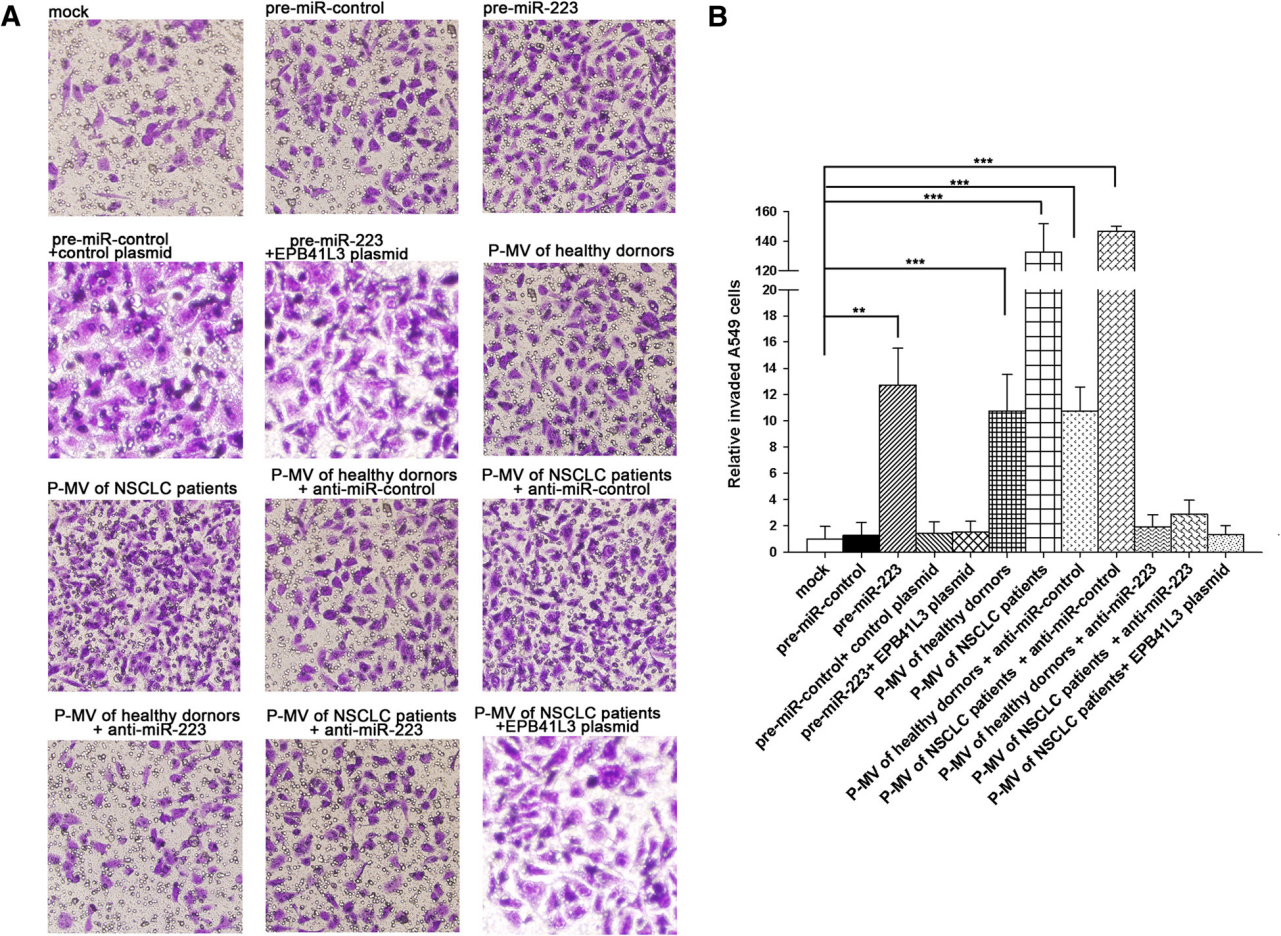
**Platelet miR-223 delivered by P-MVs promotes A549 cells migration.** **(A-** **B)** Invasion of A549 cells treated without P-MVs (mock) or with P-MVs from various sources using Transwell assay. **A**: representative image; **B**: quantitative analysis. Data are presented as the mean ± SEM of five independent experiments (*, *P* < 0.05, **, *P* < 0.01,***, *P* < 0.001).

We determined the possible role of miR-223 delivered by P-MVs targeting EPB41L3 in modulating the A549 cells invasion process. As the previous study [34], A549 cells transfected with pre-miR-223 showed an increased invasion (Figure 6A and B). Subsequently, we investigated whether overexpression or knockdown of EPB41L3 would have an impact on cell invasion. To knock down EPB41L3, a siRNA targeting EPB41L3 was designed and then transfected into A549 cells. To overexpress EPB41L3, an expression plasmid designed to specifically express the full-length open reading frame (ORF) of EPB41L3 without the miR-223-responsive 3′-UTR was also constructed and transfected into A549 cells. Efficient knockdown and overexpression of EPB41L3 in A549 cells were shown in Additional file 1: Figure S3, A-C. A549 cells transfected with EPB41L3 siRNA showed promote cell invasion; in contrast, transfection with the EPB41L3-overexpression plasmid had the opposite effect on cell invasion (Additional file 1: Figure S3, D-E). Moreover, compared with cells transfected with pre-miR-223 alone, those transfected with both pre-miR-223 and the EPB41L3-overexpression plasmid exhibited significantly lower invasion rates (Figure 6A and B), suggesting that miR-223-resistant EPB41L3 sufficiently attenuated the promotion effect of invasion of miR-223 on lung cancer cells. While P-MVs could effectively deliver miR-223 into the recipient A549 cells (Figure 3E), the invasion of A549 cells strongly increased when A549 cells incubated with P-MVs, especially, the P-MVs of NSCLC patients promoted the invasion about 100-fold compared to the P-MVs of healthy volunteers (Figure 6A and B). The promotion of A549 cells invasion by P-MVs was largely abolished by depleting miR-223 using anti-miR-223 ASO and overexpressing EPB41L3 via miR-223-resistant EPB41L3-expressing plasmid (Figure 6A and B). These results collectively showed that platelet-secreted miR-223 via P-MVs could be effectively delivered into A549 cells, in which it targets EPB41L3, and thus promotes cell invasion.

## Discussion

Thrombocytosis is frequently observed in patients with solid tumors and is associated with adverse outcomes of tumor patients. In agreement with this, we also found that PLT and PCT in NSCLC patients were significantly higher than those in cancer-free controls (Table 2). The mechanism underlying the association between thrombocytosis and tumor progression, however, remains unclear. The contribution of platelets to the pathogenesis of cancer is highly complex and dependent upon bidirectional cross-talk between platelets, tumor cells, leukocytes, stromal cells, and endothelial cells. Platelets exert their influence on tumor cells through multiple mechanisms, including direct cellular contact, local release of soluble proteins, transfer of cytoplasmic and cell surface proteins, modulation of vascular tone and permeability, activation of coagulation, and sequestration of tumor cell proteins. In addition to the physical interaction with different cells and the secretion of proteins, platelets can release a large amount of functionally active MVs, which enter the circulation, interact with other cells and affect the function of the target cells. MVs are small vesicles shed from almost all cell types under both normal and pathological conditions [7]. Platelets are major sources of MVs in peripheral bloodstream and two-thirds of peripheral blood MVs is likely derived from platelets [50]. In response to the stimuli of various inflammatory factors or under many disease states such as sepsis [8,9], thrombocytopenia [10], arterial thrombosis [11], thrombotic thrombocytopenia [12], uremia [13], malignancy [14] and rheumatoid arthritis [15], platelets release significantly more MVs. Similar to MVs derived from other cells, P-MVs bear platelet surface receptors/ligands and have the potential to selectively interact with specific target cells. Increasing evidence demonstrates that P-MVs play a role in coagulation, angiogenesis, the metastatic spread of lung cancer [9], and the immune response of hematopoietic, endothelial, and monocytic cells [16,17]. P-MVs have also been implicated in the pathogenesis of atherosclerosis as well as the regulation of angiogenesiss [16,18]. These findings suggest that platelets can affect the functional state of other cells without the physical contact through releasing P-MVs.

**Table 2:** **Clinical parameters of platelets from healthy donors and lung cancer patients**

	**Sample number**	**PLT (×10** ^**9**^ **/L)**	**PCT (%)**	**MPV (fl)**	**PDW (%)**
Healthy	20	212.53 ± 45.41	0.2095 ± 0.09	8.35 ± 1.35	14.9 ± 1.75
NSCLC	20	268.47 ± 73.62***	0.2694 ± 0.04**	9.65 ± 1.9	16.4 ± 1.50

PLT, platelets; PCT, Platelet hematocrit; MPV, Mean platelet volume; PDW, platelet distribution width; Statistical comparison was performed by using Student’s t-test (*, *P* < 0.05, **, *P* < 0.01, ***, *P* < 0.001).

In the present study, we reported that P-MVs contain various miRNAs, particularly miR-223, which displays the highest level in platelets, and these platelet miRNAs can be delivered into recipient tumor cells via P-MVs. In the recipient cells, platelet miRNAs suppress the translation of their target genes through forming RISC complex and thus change the phenotype of the cells. First, we found that the levels of miR-223 in both platelets and P-MVs were significantly upregulated in lung cancer patients compared to those in cancer-free controls. Second, *in vitro* assays with cell tracing and qRT-PCR analysis showed that platelet miR-223 was rapidly delivered into lung cancer cells via P-MVs. Third, exogenous miR-223 derived from platelets reduced the protein level of tumor suppressor EPB41L3 and thus promoted the lung cancer cells invasion. The effect of P-MVs on EPB41L3 reduction and enhancement of tumor cell invasion was largely abolished by depleting miR-223, suggesting that miR-223 play a key role in these processes. This study provides another evidence supporting the concept that cell-secreted MVs serve as physiological carriers of functional miRNAs in exchanging genetic materials and signaling molecules between cells [51,52].

As a hematopoietic-specific miRNA with crucial functions in myeloid lineage development [53,54], miR-223 has been shown to target a transcription factor Mef2c [53], CEBP-beta [55], glutamate receptors (GluR2 and NR2B) [56], IGF-1R [57] and EPB41L3 [34] in other cell types. By the luciferase reporter assay and experimental validation, we confirmed EPB41L3 as a target of miR-223 in lung cancer A549 cells. To support the role of miR-223-targeting EPB41L3 to promote A549 cells invasion, direct silencing of EPB41L3 expression by EPB41L3 siRNA showed an increased A549 cells invasion and overexpressing of EPB41L3 expression by miR-223-resistant EPB41L3-overexpress plasmid showed a suppressed A549 cell invasion (Additional file 1: Figure S3). Although the effect of factors other than platelet miR-223 on the reduction of EPB41L3 and promotion of A549 cells invasion cannot be excluded at this stage, depleting miR-223 in A549 cells by anti-miR-223 ASO largely reversed the enhancement of A549 cell invasion by P-MVs, implicating that miR-223 in P-MVs plays a key role in modulating A549 cell invasion by P-MVs. The effect of P-MVs on reducing target cell EPB41L3 and promoting tumor cell invasion is in proportion to the level of miR-223 in P-MVs. As can be seen in Figure 6, treatment with the P-MVs from NSCLC patients, which contain higher level of miR-223 than the P-MVs from cancer-free donors, resulted in the largest invasion of tumor cells.

Recent studies by others [24-30] and us [31] have shown that anucleate platelets contain large amount of miRNAs and these platelet miRNAs are clinically and biologically relevant. Interestingly, we found that, under tumor condition, the level of miR-223 in both platelets and P-MVs were increased. This is in agreement with our previous finding that platelet miRNAs are upregulated following platelet activation [31]. Although our data indicate that upregulation of miR-223 in anucleate platelets is not derived from de novo miR-223 synthetic pathway but from the direct maturation of pre-miR-223, the mechanism by which the upregulation of miRNAs in platelets is governed remains to be further explored.

## Conclusions

In summary, our results demonstrate the miR-223 level in the platelet and P-MVs were significantly increased during lung cancers patients, and that through release of miR-223-containing P-MVs, platelets of lung cancers patients can remotely modulate the invasion of lung cancer cells. Furthermore, as depletion of miR-223 by anti-miR-223 ASO and miR-223-resistant EPB41L3-overexpress plasmid treatment can effectively decreased A549 cells invasion, our study also provides a potential miRNA-based therapeutic strategy for lung cancers.

## Materials and method

### Reagents, cells and antibodies

Human lung cancer cells (A549 cells) and mouse lung cancer cells (LLC cells) were purchased from the China Cell Culture Center (Shanghai, China) and cultured in DMEM supplemented with 10% fetal bovine serum (GIBCO, Foster City, CA), all cells were incubated in a 5% CO_2_ at 37°C in a water-saturated atmosphere. Anti-EPB41L3 and anti-GAPDH antibodies were purchased from Santa Cruz Biotechnology (Santa Cruz, CA). Synthetic oligonucleotides, including pre-miR-223, anti-miR-223, and scrambled negative control (pre-miR-control and anti-miR-control), were purchased from Ambion (Austin, TX).

### Animals

Animal maintenance and experimental procedures were carried out in accordance with the US National Institute of Health Guidelines for Use of Experimental Animals and approved by the Medicine Animal Care Committee of Nanjing University (Nanjing, China). The logarithmic phase of LLC cells were collected and injected into SCID mice (the Model Animal Research Centre of Nanjing University) (1 × 10^6^ cell/per mouse) via the tail vein to establish Lewis lung carcinoma orthotopic model [58,59]. After mice were sacrificed at the end of the experiment, blood samples were collected via cardiac puncture and tumor section slides were subjected to immunohistochemical analysis using H&E staining.

### Blood collection

Patients with pathologically confirmed, newly diagnosed and untreated cancers were recruited at the Jinling Hospital (Nanjing, China). Blood samples were collected from the patients and healthy participants at the Jinling Hospital. Written informed consent was obtained from each patient and healthy participant prior to the study, and the study protocol was approved by the ethics committee of Nanjing University (Nanjing, China). The clinical features of the patients are listed in Table 1.

### Platelet isolation

Platelet-rich plasma was diluted in washing buffer (10 mM HEPES, 136 mM NaCl, 2.7 mM KCl, 2 mM MgCl_2_, 25 mM glucose, 4.2 mM EDTA, 4.2 mM trisodium citrate and 1 mM PGE, pH 6.6). The platelet suspension was centrifuged at 750 *g* for 15 min at 20°C, and the pellet was re-suspended in wash buffer without PGE for an additional centrifugation in the same conditions. Finally, platelets were recovered in suspension buffer (10 mM HEPES, 136 mM NaCl, 2.7 mM KCl, 2 mM MgCl_2_, 25 mM glucose).

### Microvesicle isolation and incubation with A549 cells

To isolate P-MVs, platelets in PRP were centrifuged at 2000 *g* for 15 min at 4°C, and the PMP-enriched plasma was collected. The plasma was centrifuged at 100,000 *g* for 1 h at 4°C in a TL-100 ultracentrifuge (Beckman Coulter). P-MVs were collected from the pellet and resuspended in FBS-free RPMI 1640 medium. For incubation of P-MVs with A549 cells, A549 cells were seeded on 12-well dishes the night before, and 200 μg P-MVs isolated from platelets were added into each well. After incubation for 24 h, A549 cells were collected for qRT-PCR and the quantitative protein assay.

### Fluorescence labeling of P-MVs for confocal microscopy

To analyze the P-MVs released from platelets under various conditions, platelets were labeled with DiI-C_16_ and then washed three times with PBS. The cells were re-suspended and the supernatant was collected and centrifuged to isolate P-MVs. P-MVs in DMEM medium were incubated with cultured A549 cells. After incubation for 12 h, A549 cells were washed, fixed, and observed under confocal microscopy (FV1000; Olympus, Tokyo). The pictures were taken under the following conditions: Objective Lens: PLAPON 60× O NA: 1.42; Scan Mode: XY; Excitation Wavelength: 405 nm for DAPI and 543 nm for DiI-C_16_; Image Size: 1024 × 1024 Pixel.

### RNA isolation and quantitative RT-PCR of miRNAs and pre-miRNA

Total RNA was extracted using TRIzol Reagent (Invitrogen). Quantitative RT-PCR was carried out using TaqMan miRNA probes (Applied Biosystems; Foster City, CA) or synthesized primer (Invitrogen). Briefly, 5 μl of total RNA was reverse transcribed to cDNA using AMV reverse transcriptase (TaKaRa; Dalian, China) and a stem-loop RT primer (Applied Biosystems). Real-time PCR was performed using a TaqMan PCR kit on the 7300 Sequence Detection System (Applied Biosystems). All reactions, including no-template controls, were run in triplicate. After the reaction, the C_T_ values were determined using fixed threshold settings. To calculate the absolute expression levels of target miRNAs, a series of synthetic miRNA oligonucleotides at known concentrations were also reverse transcribed and amplified. The absolute amount of each miRNA was then calculated by referring to the standard curve. In the experiments presented here, miRNA expression in A549 cells is normalized to U6. Because platelets are anucleate, miRNA expression in platelet cells is normalized to the total RNA sampling amount. The expression levels of target miRNAs in platelet MVs were directly normalized to the total protein content of MVs.

### Luciferase reporter assay

To test the direct binding of miR-223 to the target gene EPB41L3, a luciferase reporter assay was performed. The entire 3′-UTR of human EPB41L3 was PCR amplified from human genomic DNA. The PCR products were inserted into the p-MIR-reporter plasmid (Ambion) and the insertion was confirmed by sequencing. To test the binding specificity, the sequences that interacted with the miR-223 seed sequence were mutated (from AACUGAC to UUGACUG), and the mutant EPB41L3 3′-UTR was inserted into an equivalent luciferase reporter. For luciferase reporter assays, A549 cells were cultured in 24-well plates, and each well was transfected with 1 μg of firefly luciferase reporter plasmid, 1 μg of a β-galactosidase (β-gal) expression plasmid (Ambion), and equal amounts (100 pmol) of pre-miR-223 or the scrambled negative control RNA using Lipofectamine 2000 (Invitrogen). The β-gal plasmid was used as a transfection control. Cells were assayed using a luciferase assay kit 24 h post-transfection (Promega, Madison, WI).

### Transfection with pre-miR-control, pre-miR-223, small interference RNAs (siRNAs) and miR-223-resistant EPB41L3-expressing plasmid

A549 cells were seeded on 6-well dishes and were transfected the following day using Lipofectamine 2000 (Invitrogen). For miR-223 overexpression, 100 pM pre-miR-223 or scrambled control miRNA (pre-miR control) was used (Invitrogen). Cells were harvested 24 h after transfection. EPB41L3 siRNA was designed to target the coding region of the EPB41L3 mRNA (5′-GCUCGAAUAUCAGCAAUUA-3′). Negative control siRNAs (siRNA control), which do not lead to the specific degradation of any known cellular mRNA, were used as the negative control. A mammalian expression plasmid encoding the human EPB41L3 open reading frame (pReceiver-M02-EPB41L3) was purchased from GeneCopoeia (Germantown, MD). An empty plasmid served as a negative control. The siRNAs and plasmid were delivered into the cultured A549 cells by Lipofectamine 2000 (Invitrogen). At 24 h post-transfection, RNAs were extracted for qRT-PCR analyses and cell lysates were prepared for western blot. Cell invasion assays were performed at 24 h post-transfection.

### Invasion assay

For transwell invasion assays, 1 × 10^5^ cells were plated in the top chamber with Matrigel-coated membrane (24-well insert; 8-mm pore size; Corning Costar Corp). Cells were plated in medium without serum. Medium supplemented with serum was used as a chemoattractant in the lower chamber. The cells were incubated for 24 h and cells that did not invade through the pores were removed by a cotton swab. Cells on the lower surface of the membranes were fixed with methanol and stained with H&E.

### Western blotting

EPB41L3 protein levels were quantified by western blot analysis of whole-cell extracts using antibodies against EPB41L3. Normalization was performed by blotting the same samples with an antibody against GAPDH. Protein bands were analyzed using Bandscan software (Image J).

### Statistical analysis

All images of western blots and flow cytometry are representative of at least three independent experiments. For each experiment, qRT-PCR assays were performed in triplicate. The data are presented as the means ± SEM for three or more independent experiments. Differences are considered statistically significant at *p* < 0.05, analyzed using Student’s *t*-test.

## Additional file

Additional file 1: Table S1. PCR Primers used to amplify the human miRNAs precursors. **Figure S1**. The absolute levels of miRNAs in platelets from cancer-free volunteers and NSCLC patients detected by qRT-PCR. Results are presented as mean ± SEM of five independent experiments (*, *P* < 0.05, **, *P* < 0.01,***, *P* < 0.001). **Figure S2**. Representative H&E-stained sections of the lung tissues from the Lewis lung carcinoma mice. **Figure S3**. Downregulation of EPB41L3 by siRNA and upregulation of EPB41L3 by an overexpression vector in A549 cells. (A) Quantitative RT-PCR analysis of EPB41L3 mRNA levels in A549 cells treated with control siRNA or EPB41L3 siRNA, control plasmid or EPB41L3 plasmid. (B and C) Western blotting analysis of EPB41L3 protein levels in A549 cells treated with control siRNA or EPB41L3 siRNA, control plasmid or EPB41L3 plasmid. B: representative image; C: quantitative analysis. (D and E) Transwell analysis of A549 cells treated with control siRNA or EPB41L3 siRNA, control plasmid or EPB41L3 plasmid for 24 h. D: representative image; E: quantitative analysis. Data are presented as the mean ± SEM of five independent experiments (*, *P* < 0.05, **, *P* < 0.01,***, *P* < 0.001).

## Abbreviations

EPB41L3: Band 4.1-like protein 3
miR-223: microRNA-223
NSCLC: Non-small cell lung cancer
P-MVs: Platelet-secreted microvesicles
MVs: Microvesicles
PLT: Platelet numbers
PCT: Platelet hematocrit
MPV: The mean platelet volume
PDW: Platelet distribution width
