# Supplementary material for: MicroRNA-223 delivered by platelet-derived microvesicles promotes lung cancer cell invasion via targeting tumor suppressor EPB41L3
Source: Mol Cancer. 2015 Mar 11;14:58. doi: 10.1186/s12943-015-0327-z (PMC4360939; doi:10.1186/s12943-015-0327-z)
Supplement: Additional file 1: Table S1. — PCR Primers used to amplify the human miRNAs precursors. Figure S1. The absolute levels of miRNAs in platelets from cancer-free volunteers and NSCLC patients detected by qRT-PCR. Results are presented as mean ± SEM of five independent experiments (*, P < 0.05, **, P < 0.01,***, P < 0.001). Figure S2. Representative H&E-stained sections of the lung tissues from the Lewis lung carcinoma mice. Figure S3. Downregulation of EPB41L3 by siRNA and upregulation of EPB41L3 by an overexpression vector in A549 cells. (A) Quantitative RT-PCR analysis of EPB41L3 mRNA levels in A549 cells treated with control siRNA or EPB41L3 siRNA, control plasmid or EPB41L3 plasmid. (B and C) Western blotting analysis of EPB41L3 protein levels in A549 cells treated with control siRNA or EPB41L3 siRNA, control plasmid or EPB41L3 plasmid. B: representative image; C: quantitative analysis. (D and E) Transwell analysis of A549 cells treated with control siRNA or EPB41L3 siRNA, control plasmid or EPB41L3 plasmid for 24 h. D: representative image; E: quantitative analysis. Data are presented as the mean ± SEM of five independent experiments (*, P < 0.05, **, P < 0.01,***, P < 0.001). [file 12943_2015_327_MOESM1_ESM.doc]

**Supplementary Materials**

**Table S**1. PCR Primers used to amplify the human miRNAs precursors.

|  | | | |
| --- | --- | --- | --- |
| **miRNA** | **Forward primer (5’ → 3’)** | **Reverse primer (5’ → 3’)** | **Tm primers** |
|  |  |  | **(Forward/Reverse) °C** |
| U 6 | CTCGCTTCGGCAGCACA | AACGCTTCACGAATTTGCGT | 59/59 |
| Pre-miR-223 | CCGTGTATTTGACAAGCTGAGT | TGGGGTATTTGACAAACTGACA | 56/57 |


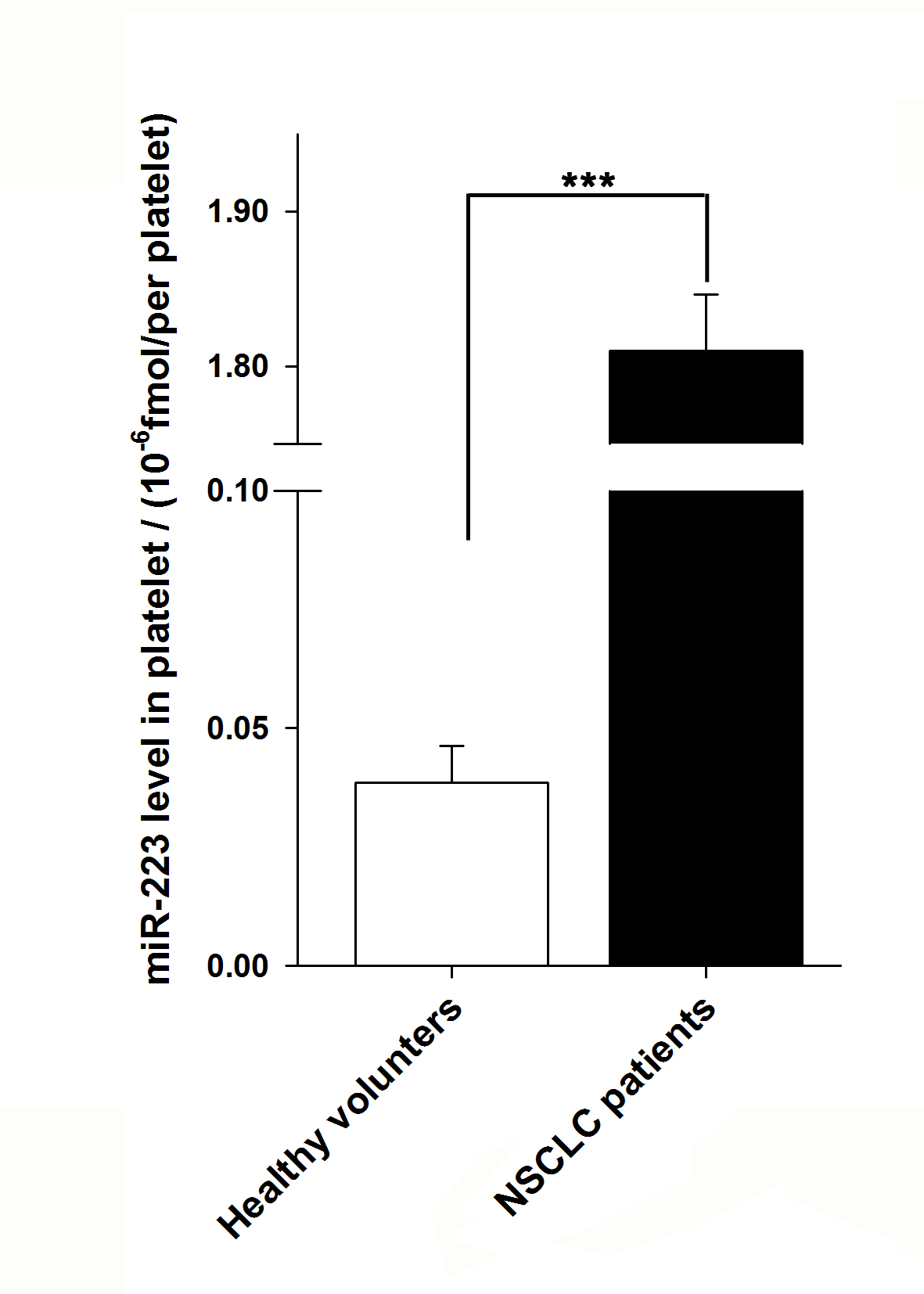


**Figure S1.** The absolute levels of miRNAs in platelets from cancer-free volunteers and NSCLC patients detected by qRT-PCR. Results are presented as mean ± SEM of five independent experiments (*, *P* < 0.05, **, *P* < 0.01,***, *P* < 0.001).


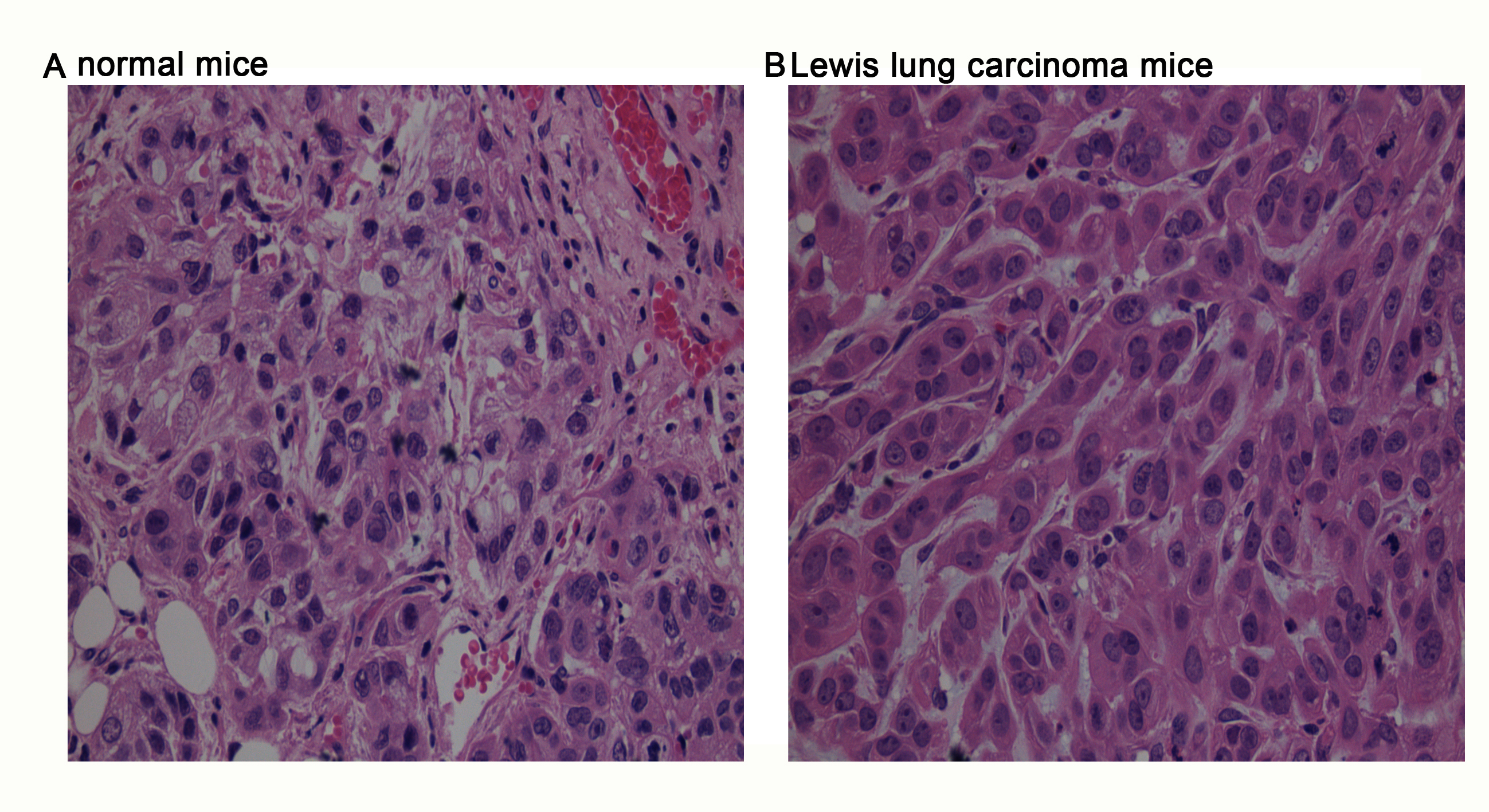


**Figure S2.** Representative H&E-stained sections of the lung tissues from the Lewis lung carcinoma mice.

**
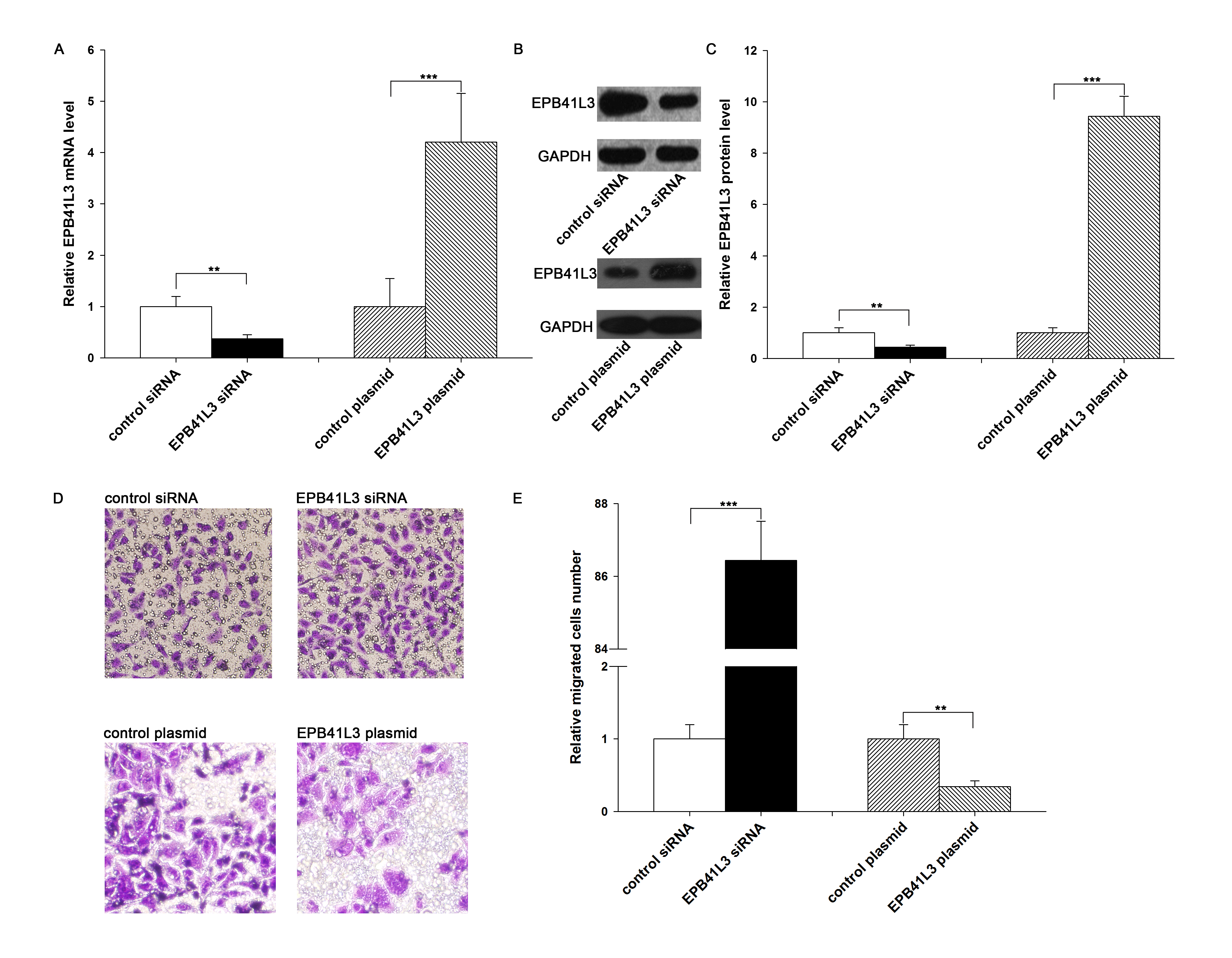
**

**Figure S3.** **Downregulation of EPB41L3 by siRNA and upregulation of EPB41L3 by an overexpression vector in A549 cells.** (A) Quantitative RT-PCR analysis of EPB41L3 mRNA levels in A549 cells treated with control siRNA or EPB41L3 siRNA, control plasmid or EPB41L3 plasmid. (B and C) Western blotting analysis of EPB41L3 protein levels in A549 cells treated with control siRNA or EPB41L3 siRNA, control plasmid or EPB41L3 plasmid. B: representative image; C: quantitative analysis. (D and E) Transwell analysis of A549 cells treated with control siRNA or EPB41L3 siRNA, control plasmid or EPB41L3 plasmid for 24 h. D: representative image; E: quantitative analysis. Data are presented as the mean ± SEM of five independent experiments(*, *P* < 0.05, **, *P* < 0.01,***, *P* < 0.001).
